# Nicotine ameliorates schizophrenia-like cognitive deficits induced by maternal LPS exposure: a study in rats

**DOI:** 10.1242/dmm.025072

**Published:** 2016-10-01

**Authors:** Uta Waterhouse, Vic E. Roper, Katharine A. Brennan, Bart A. Ellenbroek

**Affiliations:** School of Psychology, Victoria University of Wellington, P.O. Box 600, Wellington 6140, New Zealand

**Keywords:** Prenatal immune challenge, Lipopolysaccharide, Memory

## Abstract

Maternal exposure to infectious agents is a predisposing factor for schizophrenia with associated cognitive deficits in offspring. A high incidence of smoking in these individuals in adulthood might be, at least in part, due to the cognitive-enhancing effects of nicotine. Here, we have used prenatal exposure to maternal lipopolysaccharide (LPS, bacterial endotoxin) at different time points as a model for cognitive deficits in schizophrenia to determine whether nicotine reverses any associated impairments. Pregnant rats were treated subcutaneously with LPS (0.5 mg/kg) at one of three neurodevelopmental time periods [gestation days (GD) 10-11, 15-16, 18-19]. Cognitive assessment in male offspring commenced in early adulthood [postnatal day (PND) 60] and included: prepulse inhibition (PPI), latent inhibition (LI) and delayed non-matching to sample (DNMTS). Following PND 100, daily nicotine injections (0.6 mg/kg, subcutaneously) were administered, and animals were re-tested in the same tasks (PND 110). Only maternal LPS exposure early during fetal neurodevelopment (GD 10-11) resulted in deficits in all tests compared to animals that had been prenatally exposed to saline at the same gestational time point. Repeated nicotine treatment led to global (PPI) and selective (LI) improvements in performance. Early but not later prenatal LPS exposure induced consistent deficits in cognitive tests with relevance for schizophrenia. Nicotine reversed the LPS-induced deficits in selective attention (LI) and induced a global enhancement of sensorimotor gating (PPI).

## Abstract (precis)

**Summary:** This is the first pharmacological study examining the effect of repeated nicotine treatment on multiple schizophrenia-like cognitive deficits induced by maternal exposure to an infectious agent (LPS) in rats.

## INTRODUCTION

Schizophrenia develops owing to an interaction of multiple factors of genetic and environmental origin (Caspi et al., 2005; Clarke et al., 2009; Fleming and Martin, 2011; Modinos et al., 2013; Mulle, 2012). Early pre- and postnatal environmental factors are central to the neurodevelopmental theory, which argues that disturbances early during neuronal development predispose an individual to schizophrenia (for review, see Khandaker et al., 2013, 2011; Murray and Lewis, 1987). Maternal infection (for example, influenza, rubella, measles) has been identified as a prenatal risk factor, and the subsequent process of inflammation is thought to interfere with early fetal brain development, increasing the susceptibility of the offspring to later developing schizophrenia (Brown, 2012a,b; Ellman et al., 2008; Meyer and Feldon, 2009; Miller et al., 2013).

Based on findings in human research, studies have successfully utilized the model of prenatal exposure to maternal infectious agents in animals. These preclinical studies have revealed important behavioral, neurophysiological and neurochemical alterations relevant to those found in individuals with schizophrenia (for review, see Boksa, 2010; Brown and Derkits, 2010). However, a large variety of protocols to expose animals to infectious agents exist, and many different behavioral effects have been reported. For example, several different agents such as lipopolysaccharide (LPS, a bacterial endotoxin), polyinosinic:polycytidylic acid (polyI:C, a viral mimetic) or turpentine (induces local inflammation) have been used to induce maternal immune activation (MIA). Likewise, these agents have been administered at various time points during gestation using either a single injection or repeated administrations. Finally, relatively large differences in the doses have been reported. For instance, some studies have intraperitoneally injected pregnant rats on two consecutive gestational days (GD) with 0.025-0.1 mg/kg of bodyweight with LPS (Fortier et al., 2007), others have subcutaneously injected 0.5 mg/kg on GD 14, 16, 18 and 20 (Graciarena et al., 2010), or even 1 mg/kg subcutaneously every second day from GD 7 until delivery (Basta-Kaim et al., 2012).

These differences in protocol have led to varying behavioral and neurobiological effects, yet there is nonetheless substantial face validity (defined as symptom similarity between the model and the clinical condition) for schizophrenia in these models. However, much less is known with respect to the predictive validity of these models. Indeed, very few researchers have examined whether pharmacological manipulations affect alterations produced by maternal infectious agent exposure. Basta-Kaim and colleagues (2012), for example, have looked at the effect of antipsychotic medication on cognitive deficits induced by prenatal exposure to LPS (1 mg/kg, subcutaneous injections every second day from GD 7). Their findings revealed that clozapine, but not chlorpromazine, reverses deficits in sensorimotor gating (Basta-Kaim et al., 2012). Whether these data have clinical relevance is currently unknown. In general, cognitive symptoms in affected individuals are relatively unresponsive to antipsychotic medication (Ellenbroek, 2012). However, there this some evidence that sensorimotor gating might respond to (second generation) antipsychotics (for review, see Kumari and Sharma, 2002), yet longitudinal studies have provided contradictory results (Mackeprang et al., 2002).

Cognitive symptoms occur in approximately 75% of all individuals with schizophrenia. Deficits can manifest in domains such as impairments in language, attention, memory, information processing, verbal memory and executive functioning (Khandaker et al., 2013; Altamura et al., 2013; Brown et al., 2009). Cognitive dysfunctions are amongst the most debilitating and problematic symptoms as they are, as aforementioned, not very responsive to antipsychotic medication (Ellenbroek, 2012), yet they considerably impact daily functioning (Green, 1996; D'Souza and Markou, 2012). Although the treatment of cognitive deficits in schizophrenia has proven particularly difficult, there is some evidence that nicotine can reverse some of the cognitive impairments (for review, see Heishman et al., 2010). However, studies investigating the effect of nicotine in schizophrenia are confounded by the fact that the vast majority of affected individuals are (heavy) smokers (De Leon and Diaz, 2005). Animal models, however, allow the assessment of potentially beneficial effects of nicotine in drug-naïve subjects.

The aim of the present study was to determine whether repeated nicotine exposure could reverse cognitive deficits induced by maternal LPS treatment. Given the many varying existing procedures for maternal exposure to infectious agents, we first examined three different protocols to test whether these produce cognitive deficits and impairments in sensorimotor gating (similar to those found in individuals with schizophrenia) compared to controls that had been prenatally exposed to maternal saline treatment at the same time points. To this end, LPS (0.5 mg/kg of base weight, subcutaneously) was administered to pregnant rats at one of three crucial neurodevelopmental time points in fetal brain development (GDs 10-11, 15-16 or 18-19) to model insults during early, middle or late stages of rat pregnancy (Fortier et al., 2007).

To assess cognitive performance in offspring in adulthood, three specific paradigms were used that measure aspects in domains that are commonly impaired in schizophrenia and utilize comparable parameters to those used in human assessment. Firstly, deficits in selective attention (ability to ‘tune-out irrelevant information’) were assessed in latent inhibition (LI) (Gray et al., 1995; Lubow and Gewirtz, 1995; Rascle et al., 2001; Weiner and Feldon, 1997; Weiner, 2003). Individuals with schizophrenia often show a lack of LI, especially during the early stages of the illness (Lubow and Gewirtz, 1995; Weiner, 2003). Secondly, deficits in working memory, such as goal maintenance and interference control, are also frequently found in schizophrenia (for review, see Park and Gooding, 2014). Thirdly, impaired sensorimotor gating assessed in prepulse inhibition (PPI) is commonly seen in individuals with schizophrenia (Braff et al., 2001a,b; De Koning et al., 2014).

To our knowledge, this is the first study to examine the effect of repeated nicotine treatment on multiple cognitive impairments induced by prenatal exposure of rates to infectious agents.

## RESULTS

### Prepulse inhibition

**Fig. 1.**
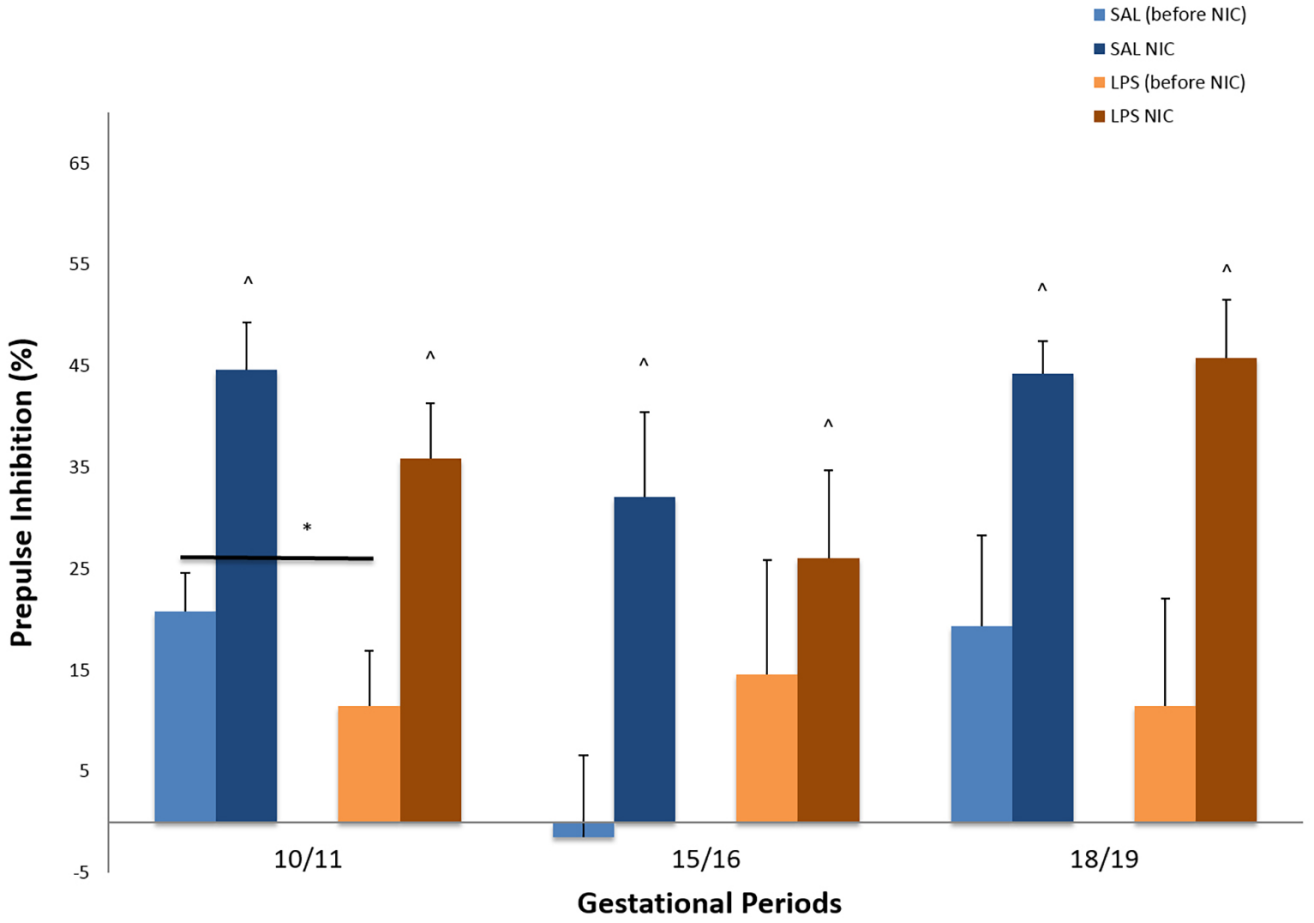
**Percent prepulse inhibition as the group average for animals prenatally exposed to maternal treatment with LPS or saline control at three gestational periods, measured at two time points – pre and post nicotine treatment.** Group sizes before and after nicotine (NIC) are the same. GD 10-11: saline (SAL), *n*=12; LPS, *n*=11. GD 15-16: SAL, *n*=11; LPS, *n*=7. GD 18-19: SAL, *n*=12; LPS, *n*=11. * indicates a significant difference for prenatal treatment (between subject factor) at gestational days 10-11 (prenatally exposed to maternal LPS versus saline; *P*<0.05, mixed ANOVA). ^ indicates a significant difference for time (within subject factor, before versus after nicotine treatment) evident at all gestational periods (GD 10-11, *P*<0.001; GD 15-16, *P*<0.003; GD 18-19, *P*=0.001, mixed ANOVA). Data are mean±s.e.m.

Prepulse inhibition (PPI) is a measure of sensorimotor gating, a filtering mechanism to protect the brain from overstimulation. It has consistently been found to be reduced in individuals with schizophrenia. Thus we expected LPS treatment to reduce PPI and nicotine to reverse this deficit. Two outliers were detected (>3 s.d. from the mean), and these were removed from the analysis. There was a significant effect of PPI (with larger prepulses leading to stronger inhibition), but none of the interactions (with either prenatal or adult treatment) were significant. All prepulses were therefore collapsed into a single mean PPI value per experimental group (Fig. 1). A mixed ANOVA analysis was performed with the within-subjects factor time (before versus after nicotine treatment), and the between-subjects factor prenatal treatment (LPS versus saline).

Statistical analysis showed that there was a significant main effect of prenatal treatment on gestational day (GD) 10-11 on PPI, (*F*_(1,19)_=5.780, *P*<0.05). Fig. 1 shows that rats that had been prenatally exposed to LPS had significantly reduced PPI values. There was no main effect of prenatal treatment on days 15-16 or 18-19.

Nicotine treatment significantly increased PPI in all pretreatment groups: GD 10-11, *F*_(1,19)_=18.125, *P*<0.001; GD 15-16: *F*_(1,15)_=12.185, *P*=0.003; GD 18-19: *F*_(1,20)_=16.244, *P*=0.001. Because there were no interactions between treatment at prenatal and adult stages, the results indicate that nicotine improved the PPI independent of prenatal treatment. This is also evident from Fig. 1, in which all the groups (saline and LPS prenatal treated) increased PPI.

There was a statistically significant difference in basal startle amplitude (see Table S3) between treatment groups before nicotine treatment for GD 10-11 (*F*_(1,20)_=21.082, *P*<0.001), but not for the other prenatal treatment periods.

### Latent inhibition

**Fig. 2.**
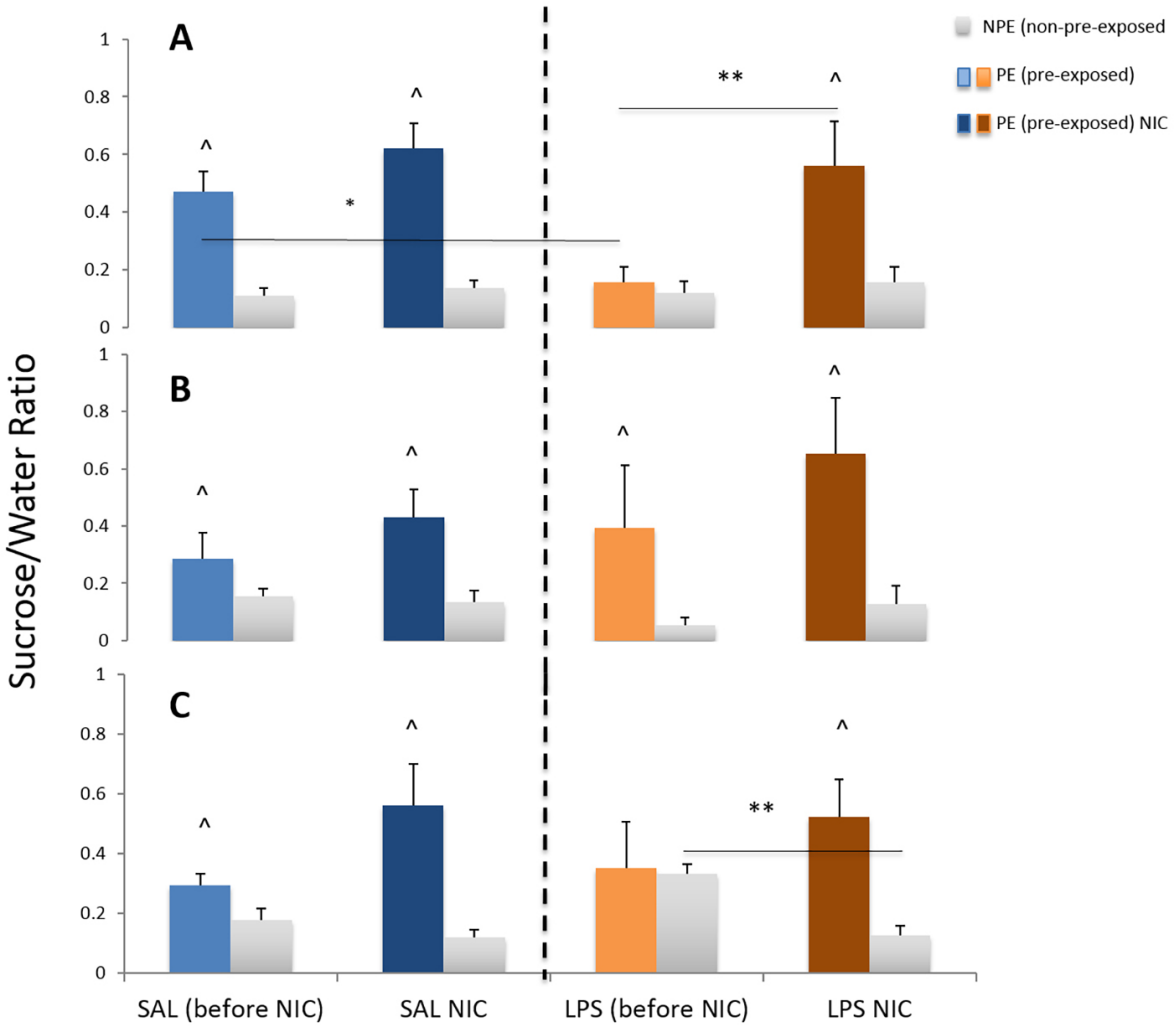
**LI as** **group average for animals that had been prenatally exposed to maternal LPS treatment or saline.** (A) GD 10-11, (B) GD 15-16 and (C) GD 18-19. LI was calculated as the consumption ratio, defined as sucrose consumption/sucrose+water consumption on testing day. LI at two time points was measured [before and after nicotine (NIC) exposure] (mean±s.e.m.). Group sizes before and after nicotine exposure are the same. GD 10-11: saline (SAL), pre-exposed, *n*=6; SAL, non-pre-exposed, *n*=6; LPS, pre-exposed, *n*=6; LPS, non-pre-exposed, *n*=5. GD 15-16: SAL, pre-exposed, *n*=6; SAL, non-pre-exposed, *n*=5; LPS, pre-exposed, *n*=3; LPS, non-pre-exposed, *n*=4. GD 18-19: SAL, pre-exposed, *n*=7; SAL, non-pre-exposed, *n*=5; LPS, pre-exposed, *n*=5; LPS, non- pre-exposed, *n*=6. ^ indicates a significant difference for LI after pre-exposure at all gestational time points between subject factor, pre-exposure with sucrose versus non-pre-exposure with sucrose. GD 10-11, *P*<0.001; GD 15-16, *P*<0.005; GD 18-19, *P*<0.001; mixed ANOVA). * indicates a significant interaction between prenatal treatment (between subject factor, maternal LPS treatment versus saline) at GD 10-11 (A) in LI with pre-exposure (mixed ANOVA, *P*<0.02). LI occurred in the SAL pre-exposed group but not in the LPS pre-exposed group. At GD 10-11 (A), ** indicates a significant effect of time (before versus after treatment with nicotine, mixed ANOVA, *P*<0.005) where nicotine normalized LI in the LPS pre-exposure group but had no effect on the LPS non-pre-exposed group. At GD 18-19 (C), ** indicates a significant interaction between LI non-pre-exposure and time (mixed ANOVA, *P*<0.03). Nicotine normalized the failed conditioning of the LPS non-pre-exposure group.

Latent inhibition (LI) is generally regarded as a model of selective attention. As the pre-exposed group has previously been presented with sucrose, attention for this stimulus has waned. As a result, conditioning of the sucrose stimulus to the aversive effects of LiCl is reduced. Thus, we expect the control pre-exposed groups to drink more sucrose than the non-pre-exposed groups. However, studies have shown that individuals with schizophrenia have reduced LI (Lubow and Gewirtz, 1995) and thus, we expect the prenatal LPS-treated group(s) to show reduced intake only in the pre-exposed groups, as they do not habituate to the sucrose stimulus during pre-exposure. In addition, we expect nicotine treatment to improve this deficit by selectively increasing consumption in the pre-exposed group. The results of the LI experiments are shown in Fig. 2A-C. A mixed ANOVA analysis was performed. The within-subjects factor was time (before versus after nicotine treatment) and the between-subjects factors were (1) treatment at prenatal stage (LPS versus saline) and (2) LI pre-exposure (sucrose versus water).

Statistical analysis revealed that, for all prenatal periods, there was a significant effect of pre-exposure: GD 10-11: *F*_(1,19)_=26.370, *P*<0.001; GD 15-16: *F*_(1,13)_=15.345, *P*<0.005; GD 18-19: *F*_(1,19)_=19.441, *P*<0.001. This shows that LI occurred where the pre-exposed animals drank considerably more sucrose on the test day than non-pre-exposed rats. However, in the GD 10-11 group, there was a significant interaction between prenatal treatment and pre-exposure: *F*_(1,19)_=7.317, *P*<0.02, but there was no three-way interaction. Inspection of data in Fig. 2A reveals that LI occurred in the prenatal saline-treated group but not in the prenatal LPS-treated group (maternal LPS exposure), owing to a substantially diminished effect in the LI pre-exposed group.

There was no significant interaction between prenatal treatment and pre-exposure in the GD 15-16 group (see Fig. 2B), whereas for the GD 18-19 group, there was again a significant interaction: *F*_(1,19)_=5.634, *P*<0.03. Interestingly, inspection of Fig. 2C shows that in contrast to prenatal treatment with LPS on GD 10-11, exposure on GD 18-19 increased sucrose intake in the non-pre-exposed group, suggesting a reduction in conditioned taste aversion.

On GD 10-11, there was a main effect of nicotine treatment: *F*_(1,19)_=11.501, *P*<0.005. Nicotine treatment restored LI in the prenatal LPS group (see Materials and Methods for explanation) by increasing the sucrose consumption in the pre-exposed group; however, the non-pre-exposed group were unaffected by treatment with nicotine.

Nicotine did not alter sucrose intake in any of the groups on GD 15-16, yet there was a significant interaction on GD 18-19 between nicotine treatment and pre-exposure (*F*_(1,19)_=5.634, *P*<0.03), but no three-way interaction. Sucrose consumption decreased in the non-pre-exposed animals in the prenatal LPS group after nicotine exposure, thus nicotine ameliorated the deficit in conditioning induced by exposure to maternal LPS treatment.

### Delayed non-matching to sample

**Fig. 3.**
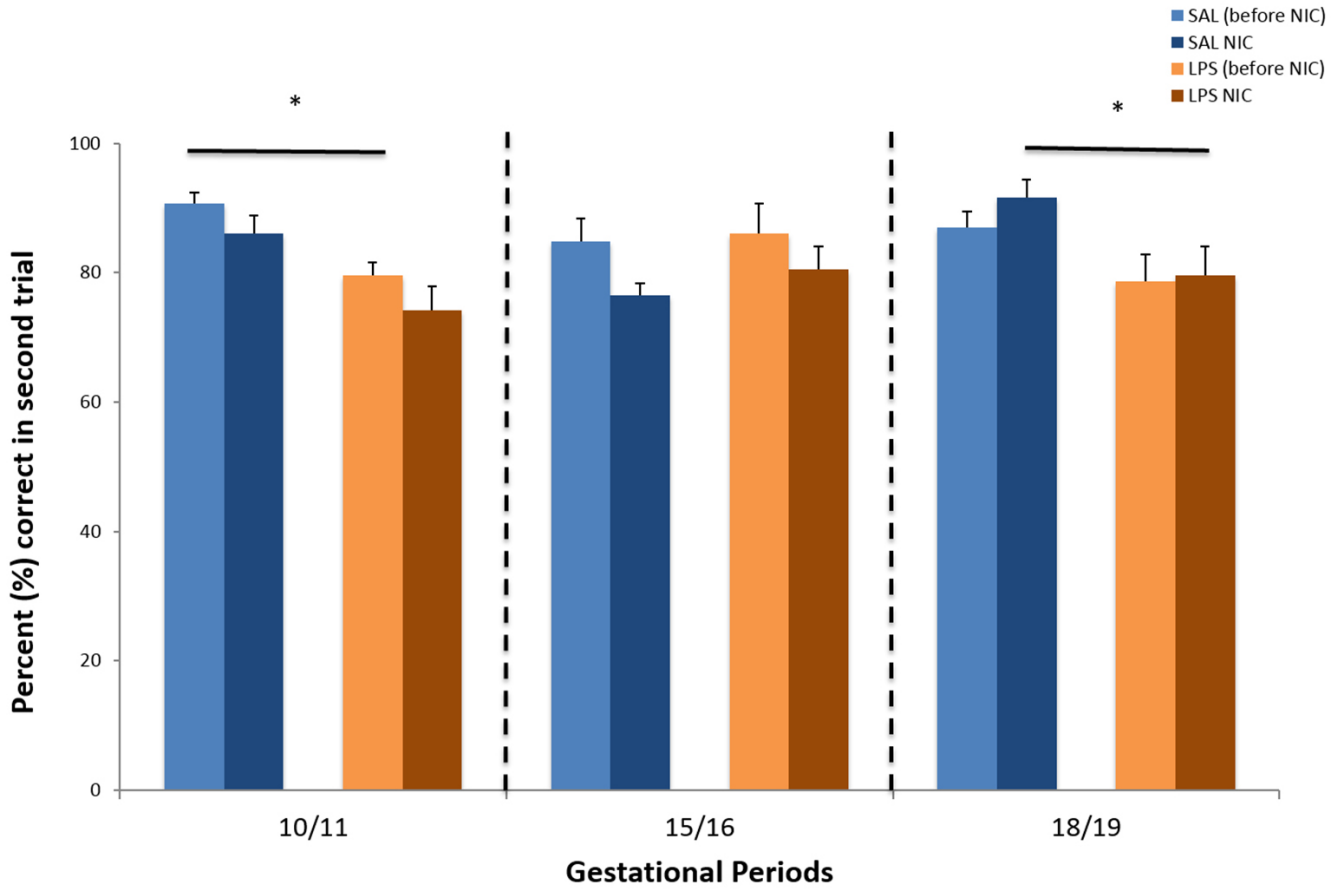
**Delayed non-matching to sample for three gestational periods.** All data are given as mean values (mean±s.e.m.) of the percent (%) correct second arm visits over four trials per day on three consecutive days. Group sizes before and after nicotine (NIC) are the same. GD 10-11: SAL, *n*=12; LPS, *n*=11. GD 15-16: SAL, *n*=11; LPS, *n*=7. GD 18-19: SAL, *n*=12; LPS, *n*=11. * indicates a significant difference for prenatal treatment (between subject factor, maternal LPS treatment versus saline) at GD 10-11 before nicotine treatment (mixed ANOVA, *P*<0.005). At GD 18-19, * indicates a significant difference for prenatal treatment after nicotine treatment (*P*<0.01).

The delayed non-matching to sample (DNMTS) test assesses working memory. Rats are required to remember a previously visited arm in order to obtain a reward in the opposite arm. With increasing intervals, the demand on working memory increases and the task becomes progressively more difficult. As individuals with schizophrenia have substantial working memory deficits, we expect prenatal LPS-treated animals to make more working memory errors than controls. In addition, we expect nicotine treatment to improve the deficit. A mixed ANOVA was performed with the within-subjects factor time (before versus after nicotine treatment) and the between-subjects factor was prenatal treatment (LPS versus saline). There was a significant effect of pretreatment on accuracy for GD 10-11: *F*_(1,18)_=14.749, *P*<0.005. Inspection of Fig. 3 revealed that the LPS group GD 10-11 made significantly more errors than the saline controls. No statistically significant effect was found in any of the other pretreatment groups.

In contrast to the previous two tests, there was no significant effect of nicotine in any of the pretreatment groups (Fig. 3). However, a statistically significant effect of pretreatment on accuracy was evident for GD 18-19 after nicotine exposure (*F*_(1,16)_=9.309, *P*<0.01). Inspection of data in Fig. 3 shows that animals that had been prenatally exposed to LPS made more accuracy errors compared to the saline-treated animals after nicotine exposure.

## DISCUSSION

The present study led to several main findings. First of all, prenatal exposure to maternal LPS treatment on GD 10-11 led to significant deficits in all tests compared to saline controls: reduced PPI, LI and delayed non-matching to sample (DNMTS). Maternal LPS administration later in development was ineffective in this respect. Secondly, specific cognitive deficits (LI) induced by maternal LPS exposure were ameliorated by repeated nicotine exposure. Finally, a general cognitive-enhancing effect of nicotine was observed in PPI in all treatment groups.

### The effects of prenatal exposure to maternal LPS treatment

The present findings were inherently consistent with those of previous studies with respect to saline controls; deficits were repeatedly found in the early-intervention group (GD 10-11). Similar to individuals with schizophrenia, animals that had been prenatally exposed to maternal LPS treatment at GD 10-11 showed impairment in PPI. These findings are also consistent with previous studies that have shown impairments in sensorimotor gating after prenatal immune activation (Borrell et al., 2002; Romero et al., 2010, 2007), although some differences have been found in relation to timing (Fortier et al., 2007). Using a similar two-day LPS protocol, Fortier and colleagues (2007) found a significant reduction in PPI after LPS exposure on GD 15-16 and 18-19, but not on GD 10-11. Methodological differences, such as the dose of LPS and the route of administration, might explain the discrepancies. In the present study, 0.5 mg/kg of LPS was subcutaneously injected, whereas Fortier et al. administered 0.025, 0.05 and 0.1 mg/kg intraperitoneally. Fortier et al. report that all pups in the GD 10-11 (but not in the GD 15-16) group died at the dose of 0.1 mg/kg, indicating that the GD 10-11 pups are very sensitive to maternal LPS exposure. Moreover, considering that we found no evidence of reduced litter size, the subcutaneous-injection protocol might have induced more subtle deficits. Compared to prenatally treated saline controls, maternal LPS exposure at later times during development did not lead to significantly reduced PPI, although the controls in the GD 15-16 group showed a relatively low PPI value.

Consistent with the PPI findings, a reduction in LI after maternal LPS exposure on GD 10-11 was found compared to saline treated controls on GD 10-11. These results could relate to individuals with schizophrenia, as this deficit in LI was specifically found in the pre-exposure group. Interestingly, maternal LPS exposure on GD 18-19 also reduced the difference between the pre-exposure and the non-pre-exposed group, yet, in this case, it was due to a selective alteration in the non-pre-exposed group. This suggests a reduced conditioned taste aversion, although as is usual for LI experiments, we did not include a LiCl-free condition. Compared to those investigating effects on PPI, fewer studies have investigated the effects of maternal infectious agent exposure on LI. However, the rodent studies published thus far are consistent with our finding that early (Meyer et al., 2005; Smith et al., 2007) but not late (Bitanihirwe et al., 2010) exposure reduces LI.

Finally, our results show that prenatal exposure to maternal LPS treatment led to a small but significant increase in errors in DNMTS, the effects again limited to the GD 10-11 group and could relate to several studies that show deficits in working memory in individuals with schizophrenia (Park and Gooding, 2014). Several studies have found deficits in cognitive performance after maternal immune challenge, such as deficits in the novel object recognition test (Graciarena et al., 2010) or spatial learning (Hao et al., 2010), but to our knowledge this is the first rat study investing working memory deficits.

### Mechanisms underlying the effects of prenatal exposure to maternal LPS treatment

Our results show that prenatal exposure to maternal LPS treatment induced long-lasting cognitive deficits similar to those seen in schizophrenia. These results are largely congruent with those of previous studies using LPS or other immune-activating agents, such as polyI:C or turpentine. Because these different agents appear to induce comparable deficits in offspring (Boksa, 2010; Meyer, 2014), it seems likely that they all affect a common process. The most parsimonious common process is general activation of the maternal immune system, especially as most agents, including LPS, cannot cross the blood-placental barrier (Ashdown et al., 2006; Oskvig et al., 2012). The maternal immune response involves the production of several different pro-inflammatory cytokines in both the mother and the fetus (Meyer et al., 2009; Boksa, 2010). Prenatal exposure to the cytokine interleukin-6 (IL-6, at GD 12.5), for example, results in behavioral (PPI, LI) as well as transcriptional deficits in offspring in mice (Smith et al., 2007). Likewise, the effects of polyI:C are significantly reduced in *Il6*-knockout mice (Smith et al., 2007).

Cytokines not only play a vital role in the immunological response to infection given that they subsequently lead to the eradication of foreign infectious agents (Curfs et al., 1997) but are also involved in many aspects of normal brain development, including neurogenesis and synaptogenesis (Howard, 2013; Meyer et al., 2009). Thus the ‘cytokine hypothesis’ states that elevated cytokine levels that are induced by an immune challenge during gestation interfere with fetal brain development. Consistent with changes in brain morphology (Li et al., 2009; Cui et al., 2009; Graciarena et al., 2010), alterations in neurotransmitter transmission (Boksa, 2010) have been reported, as well as alterations in cell migration and synapse maturation (Cui et al., 2009). For example, maternal immune activation early during gestation (GD 9.5) leads to reductions in dopamine and dopamine metabolite levels in the striatum (Soto et al., 2013; Kirsten et al., 2012, 2010), as well as to abnormalities in dopamine and serotonin activity in the substantia nigra (Wang et al., 2009). These early alterations might affect adult brain functioning, leading to abnormalities in dopamine signaling (Eyles et al., 2012). The crucial role of dopamine in cognitive performance has been well established in PPI (Ellenbroek et al., 1996; Mosher et al., 2015) and LI (Diaz et al., 2015), as well as working memory (Cools, 2011; Cools and D'Esposito, 2011).

The development of the brain and specific brain structures is sequential (Workman et al., 2013), explaining why timing of the maternal infection is crucial for the long-term outcome, as is evident in the present and other studies (Meyer et al., 2007; Fortier et al., 2007). Which brain regions are crucially altered in the early (GD 10-11) but not the later gestational period and how these regions are linked to the cognitive deficits observed in the present study remains to be investigated.

### The effects of nicotine exposure

So far, the studies modeling maternal infectious agent exposure in rodents have primarily focused on the face validity; for example, does the model lead to abnormalities similar to those seen in schizophrenia? Very few studies have investigated whether pharmacological treatment can reduce these deficits. As mentioned previously, clozapine but not chlorpromazine reverses the effects of repeated gestational LPS treatment on PPI (Basta-Kaim et al., 2012). Likewise, in the only study examining the effects of nicotine in rodent models of infectious agent exposure, a single acute injection of nicotine reverses the effects of neonatal exposure to polyI:C (5 mg/kg, subcutaneous, postnatal days 2-6) on their object recognition ability, but not in PPI (Yu et al., 2010). Because nicotine has a relatively short half life, and repeated treatments are required to observe its full effect, we studied the effects of repeated nicotine exposure on LPS-induced cognitive deficits. The results revealed that nicotine, as predicted, improved performance, although intriguing differences were found between the three paradigms. With respect to PPI, nicotine induced a global increase in PPI, independent of prenatal treatment. Thus, not only was the GD 10-11 deficit ameliorated, but also the normal performance of rats that had been prenatally treated with saline (or LPS on days 15-16 or 18-19) was enhanced. Similar global increases in PPI have been reported in humans, in both healthy volunteers and individuals with schizophrenia (Kumari and Postma, 2005; Levin et al., 2006; Newhouse et al., 2011). However, not all studies have shown an improvement by nicotine on PPI. As aforementioned, Yu et al. (2010) found no enhancing effect of nicotine (0.15 or 0.5 mg/kg, subcutaneously) in mice that had been prenatally exposed to maternal infectious agents. However, a single injection of nicotine was administered to model an acute effect as opposed to repeated nicotine treatment, which leads to different neurochemical alterations (Brennan et al., 2010), and therefore might affect cognitive performance differently.

Consistent with the findings for PPI, a trend for nicotine-induced increased LI was observed in all sucrose pre-exposure groups, yet this was not statistically significant. Additional experiments, for example, with reduced pre-exposure, are necessary to explain this phenomenon. However, nicotine treatment restored the LI that was disrupted by treatment with LPS on GD 10-11 by selectively increasing the sucrose consumption in the pre-exposed group. Thus, a more specific effect of nicotine was found in this paradigm. Because LI has been directly linked to selective attention, these data are consistent with studies in humans (Bates et al., 1995), where nicotine had a stronger effect in individuals with impairments in selective attention compared to those that display normal levels of attention (Hahn et al., 2012; Smucny et al., 2015). A significant reduction in conditioned taste aversion was observed in the group prenatally exposed to maternal LPS treatment on GD 18-19, a phenomenon that was also reversed by the administration of nicotine. Thus, repeated nicotine exposure reversed both the decreased sucrose intake in pre-exposed animals (GD 10-11) and the increased intake in non-pre-exposed animals (GD 18-19). Because it did not significantly affect sucrose consumption in any of the other groups, the effect of nicotine on LI appeared to be specific for the LPS-exposed groups.

Finally, there was no change in performance on the DNMTS test, even in the group with a reduced performance (LPS on GD10-11) despite evidence that nicotine can improve working memory performance (for review, see D'Souza and Markou, 2012). Although Yu et al. (2010) have found a dose-dependent nicotine-induced enhancement in object recognition memory, it should be noted that object recognition is regarded more as a model for episodic memory than working memory (Levin et al., 1997).

A possible explanation for the absence of a nicotine-induced enhancement in the present study could be the presence of a ceiling effect. As with LI, it has been suggested that improvements in memory as a result of nicotine are more pronounced in subjects with a lower baseline (Niemegeers et al., 2014). Although some studies report improvements in domains such as learning, memory and attention in healthy volunteers (McClernon et al., 2003; Rusted et al., 2005), others show a more beneficial and pronounced effect of nicotine in individuals with a reduced baseline performance, such as that presented by schizophrenic individuals (Jacobsen et al., 2004; Myers et al., 2004; Jubelt et al., 2008). Accuracy of choice in the present study for those treated on GD 10-11 was 79% for the treatment group and 90% for the control group. Performance at this level could be difficult to improve. By contrast, we found a small but significant increase in performance in the GD 18-19 group (from 87 to 91% upon nicotine treatment). Thus, it remains to be investigated whether the lack of effect in the GD 10-11 group could be due to a ceiling effect. One possible way to assess this would be to increase the inter-trial intervals, which would lead to an increased demand on working memory components and reduced accuracy.

### Mechanisms underlying the effects of nicotine

The underlying mechanisms of how nicotine affects cognitive functioning remain largely elusive. Nicotine rapidly crosses the blood-brain barrier (10 to 20 s) and binds to nicotinic acetylcholine receptors (nAChRs) (Benowitz, 2009), most predominantly to the α7 and α4β2 subtypes. The importance of the cholinergic system and nAChRs in mediating cognitive processes has been observed across multiple species (Klinkenberg et al., 2011; Wallace and Porter, 2011; Rezvani and Levin, 2001), and improvements in humans have been shown in domains relevant to schizophrenia, such as sensorimotor gating (Postma et al., 2006; Woznica et al., 2009), attention (Barr et al., 2008; Hahn et al., 2012), memory (Jubelt et al., 2008) and executive control (Petrovsky et al., 2013). Neuronal α7 receptors, for example, exist at pre- and post-synaptic locations; thus, they can rapidly mediate synaptic transmission and plasticity, as well as neurotransmitter release that is relevant to cognitive functioning, such as that of acetylcholine, dopamine, glutamate, serotonin and GABA (Benowitz, 2009; Brennan et al., 2010).

The presence of these receptors on cholinergic as well as on dopaminergic neurons in brain areas such as the pre-frontal cortex highlights their importance in modulating a wide range of neurotransmitters that are crucial to cognition (for review, see Wallace and Porter, 2011). This is important as prenatal exposure to maternal infectious agents, as aforementioned, has been implicated in long-lasting alterations in most neurotransmitter levels, but in particular in dopamine. Changes in gene expression responsible for the induction and specification of dopaminergic neurons have also been identified (Eyles et al., 2012).

The α7 subtype has received great attention as a possible drug target to improve cognitive functioning (Keefe et al., 2013). Interestingly, many individuals with schizophrenia show reduced nAChRs levels (such as α7), in particular in brain areas associated with cognitive processing, presumably owing to genetic anomalies (for example in *CHRNA7*) (Freedman, 2014). For these reasons, it has been suggested that nicotine from cigarette smoke might lead to an optimization of neuronal activity levels in these brain regions by stimulating nAChRs, consequently leading to a beneficial and pro-cognitive effect in schizophrenic individuals.

### Limitations and future research

The present study demonstrates that, compared to saline controls, maternal LPS treatment produces cognitive deficits in offspring, reminiscent of schizophrenia, when the animals were exposed at GD 10-11. However, behavior was only assessed at a single time point in adulthood (postnatal day 60). Given that the symptoms of schizophrenia typically develop after puberty (although this might be less evident for the cognitive symptoms), the model could be expanded by additional behavioral analysis earlier in life (i.e. before the period of postnatal days 35-45, which marks the onset of puberty in rats). Additionally, the assessment of other aspects of schizophrenia, such as positive or negative symptoms in the maternal infectious agent exposure model, would be of interest, particularly if the timing of the infection during pregnancy affects the development of positive, negative and cognitive symptoms differently.

The main conclusion of this study is that nicotine treatment improves cognitive performance. However, the specific effects depend on the test: nicotine increased PPI in all animals (controls and those exposed to LPS), whereas it only reversed the LPS-induced deficits in LI without altering behavior in control animals. Although the current study did not include a saline-exposed control group when examining the effect of nicotine, as this would have exponentially increased the number of animals utilized to fulfill the minimum group size requirements, we have since replicated our findings that prenatal exposure to maternal LPS treatment (GD 10-11) leads to similar deficits, which did not change after saline treatment (U.W., K.A.B. and B.A.E., unpublished data). Thus, these data confirm that prenatal maternal LPS leads to long-lasting deficits in these domains and that the enhancing effect observed can be attributed to nicotine exposure.

Additionally, the present study used experimenter-administered nicotine injections to evaluate a cognitive-enhancing effect of nicotine on LPS-produced deficits. Because many individuals with schizophrenia are heavy smokers, it has been hypothesized that this could be partly due to the self-medicating properties of nicotine (for review, see Kumari and Postma, 2005; Levin, 2013), and the present data support this hypothesis. However, it is well established that self-administering drugs produce differential neurological effects to those of non-contingently (experimenter) administered drugs (Chen et al., 2008; Hemby et al., 1997). For these reasons, an extension to the present work would be to investigate the effects of nicotine self-administration on cognitive deficits in the LPS animal model for schizophrenia.

## MATERIALS AND METHODS

### Subjects

Subjects were male Sprague Dawley rats that were exposed to either prenatal maternal infectious agent exposure (*N*=29, eight litters) or saline (*N*=35, eight litters). The pregnant females used to breed these subjects were randomly assigned to one of the six different treatment conditions (either LPS or saline injections at one of three gestational periods). All animals (pregnant dams and their male offspring) were bred in the vivarium at Victoria University of Wellington. Animals were weaned at postnatal day 21 and housed in groups of two to four with unlimited access to food and water in housing facilities on a 12-h light-dark cycle (lights on at 07:00, lights off at 19:00). Rooms were controlled for humidity (77%) and temperature (21°C). From postnatal day 50 onwards, and for the duration of the experiment, the animals were housed in facilities with a reversed light-dark cycle (light: 19:00-07:00) and placed on a food-restricted diet (to reach 85-90% of their normal body weight). Animals were fed approximately 20 g of food pellets (Diet 86, Sharpes Stockfeeds, Carterton, New Zealand) per day following testing. This mild food restriction maintains a healthy gradual weight gain over time and has been previously successfully utilized in our laboratory and others to facilitate learning (Brennan et al., 2015). No statistically significant differences between treatment groups were observed in litter size and birth weight or in adult weight throughout the experiment. Cognitive and behavioral testing commenced around postnatal day 60. For a timeline of the experimental design, refer to Table S1. All methods and procedures used in this study are in accordance with the appropriate guidelines and have been approved by the Victoria University of Wellington Animal Ethics Committee (reference number AEC2013-R7).

### Drug treatments

#### Prenatal treatment

Pregnant female rats were administered a once daily injection on two consecutive days of either LPS (0.5 mg/kg, subcutaneously) or saline at one of the following gestational periods: GD 10-11, 15-16 or 18-19. This moderate LPS dose was chosen as it has previously led to (a) persistent microglial activation and (b) a down-regulation of transforming growth factor (TGF-β1) especially in the hippocampus, leading to a long-lasting reduction in cell proliferation and neurogenesis (Graciarena et al., 2010). Subjects for the present study were male offspring born to these dams. This group will also be referred to as ‘LPS group’ for simplification purposes, although offspring have not been directly exposed to the endotoxin LPS (prenatal exposure through maternal LPS treatment). For group size information, refer to Table S2.

#### Nicotine treatment

At around postnatal day 100, all animals received experimenter-administered injections of nicotine (0.6 mg/kg, subcutaneously, and dose refers to the base weight) once daily for ten consecutive days before re-testing commenced, where daily nicotine injections continued during the re-testing phase and were administered 30 min before testing. This nicotine dose was based on the observation that rats readily self-administered 0.5 to 1 mg/kg in a 2-h session (Brennan et al., 2013, 2015) in our laboratory. In addition, this dose had an ameliorating or decreasing effect on cognitive deficits in a dose-response study within our laboratory (unpublished data).

### Apparatus and procedures

#### Cognitive and behavioral tests

As some of the cognitive and behavioral tests required days or weeks of training or testing, paradigms were administered in a random and counter-balanced order during re-testing (commencing on postnatal day 110) to account for differences in nicotine levels and age differences. The minimum average time difference between the test and re-test was 40 days. Testing occurred between 09:00 and 17:00, Monday to Sunday. Each test session began with a general habituation period to the experimental room of 15 min.

#### PPI

Average startle response (ASR) and PPI of acoustic startle were assessed using four startle chambers (San Diego^®^ Instruments, San Diego, USA), for more details refer to Ellenbroek et al. (2001). Startle trials (postnatal day 120) consisted of a single acoustic burst of white noise (120 dB, 20 ms). Pre-pulse trials included prepulses with different intensities (72 dB, 74 dB, 78 dB or 86 dB) of white noise for a duration of 20 ms followed by a startle trial after a latency of 100 ms. Each session started with five startle trials, followed by ten blocks of startle and PPI trials and finished with another block of five startle trials. Percent PPI (% PPI) was calculated as follows: [1−(startle amplitude on prepulse trial/mean startle amplitude on startle trial)]×100.

#### LI

LI was assessed using the conditioned taste aversion paradigm. Within groups, animals were randomly assigned to a pre-exposure or non-pre-exposure group and were water deprived for 23.5 h before the start of the experiment (Ellenbroek et al., 1996). Animals assigned to the pre-exposure group had once daily (3 days) access for 30 min to a drinking bottle with 100 ml of a 5% sucrose solution; the non-pre-exposure animals had access to a drinking bottle containing plain water. During conditioning, all animals had access to a bottle filled with the sucrose solution for 30 min, following an injection of LiCl (75 mg/kg; in 10 ml/kg, intraperitoneally) to induce conditioned taste aversion. The following day (testing day) all animals had access to both, a bottle with 5% sucrose solution and plain water. The ratio of sucrose water consumption was calculated as follows: day 5 sucrose consumption/(day 5 sucrose+water consumption) (Ellenbroek et al., 1996).

#### DNMTS

DNMTS was assessed using a T-maze with the following dimensions: arm length 30 cm, width 9 cm and wall height 10 cm. Animals were habituated to the maze and familiarized with retrieving sugar pellets from both arms before training commenced. Once the animal had reached an accuracy of 75% correct second arm visits on three consecutive days during training, working memory testing commenced. During working memory assessment, four sessions with two trials each for three consecutive days were performed. The inter-trial intervals varied (5, 30, 60 and 120 s) and the sequence was randomly assigned over the four sessions per day with the restriction that every interval was selected once per day per animal. The variable of interest was the percent (%) of correct initial arm visits in trial 2.

### Drugs

(−)-Nicotine hydrogen tartrate salt and LPS (*Escherichia coli* 0111:B4) were obtained from Sigma-Aldrich and dissolved in 0.9% sterile saline. The nicotine solution was adjusted to pH 7.2-7.4 with NaOH. LiCl was obtained from SciChem (Bilston, West Midlands, UK) and dissolved in 0.9% sterile saline.

### Statistical analysis

Data analysis was performed using mixed analysis of variance (ANOVA) where gestational periods were considered separately, with *P*-values<0.05 considered to be statistically significant. For a detailed explanation of the within- and between-subjects factors per paradigm, please refer to the individual tests in the Results section.
